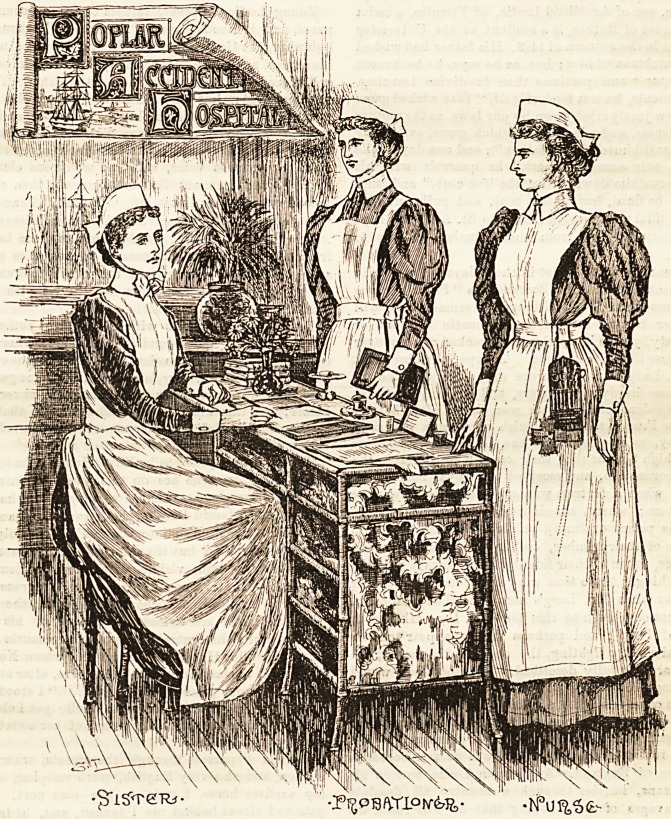# "The Hospital" Nursing Mirror

**Published:** 1896-04-11

**Authors:** 


### "The Hospital" Nursing Mirror.

Being the Extra Nursing Supplement of "The Hospital" Newspaper.

[Contributions for this Supplement should be addressed to the Editor, The Hospital, 428, Strand, London, W.C., and should have the word "Nursing" plainly written in left-hand top corner of the envelope.]

### News from the Nursing World.

### NEW HOMES FOR NURSES.

A better sense of what is due to nurses in the matter of accommodation seems to be gradually dawning on hospital committees and the public at large, and from several quarters come the welcome news that proper buildings are to be provided for nurses who have been so far but badly off in this respect. At Worcester the necessity for a nurses' home for the infirmary staff was urged at the recent annual meeting by the chairman and other speakers, and a resolution was passed to the effect that the committee would vote the sum of £2,500 if a similar amount were subscribed by the public. No doubt a liberal response will be received and immediate steps taken to provide fitting accommodation for the nurses. At Dundee active preparations are on foot for the erection of the new nurses' home for the Royal Infirmary, which is to be begun at once, thanks to the generous donation of £5,000 received from Mr. W. O. Dalgleish for that express purpose. At the Leeds General Infirmary new accommodation for the nursing staff is badly needed, and is to be taken in hand as soon as possible. An administrative block and new nurses' home are about to be provided at the West Heath Infectious Hospital, Birmingham, and in London new homes are nearly completed at the St. George's and London Hospitals, and at Chelsea Infirmary, and a similar building is to be provided at the Brompton Consumption Hospital.

### TOYNBEE NURSING GUILD.

By the invitation of Canon and Mrs. Barnett the members of the Toynbee Nursing Guild met for a social and business gathering on March 24th at the Warden's Lodge, Toynbee Hall. The guild numbers some fifty-six ladies, and was recently started with a view to continuing the instruction gained in ambulance and nursing classes. Monthly lectures have been given, and weekly bandage practices held. It is proposed, we understand, later on—when the members have become more efficient—to register a list of those "willing to give temporary assistance in sick visiting under the direction of existing nursing agencies." Particulars can be obtained from Miss Wills, hon. secretary, or from Mr. W. H. Whinny, hon. secretary, Ambulance Work, Toynbee Hall, E.

### NEW ASYLUM, GARTLOCH, N.B.

The magnificent new asylum in course of erection at Gartloch, by the Glasgow Board of of Lunacy, will be opened in a few months from this date. It is a very interesting building, for it embodies the most up-to-date treatment of the insane, and is as far removed from the asylum of the penny dreadful and the popular idea as can well be imagined. Bolts and bars are conspicuous by their absence, and the arrangements everywhere are with a view of making the inmates feel that "they are in a home to be cured, not in a prison to be coerced." The hospital is a separate building isolated from the main block, and will provide accommodation for 160 patients. It is intended to employ women nurses only in the hospital, and these are to be largely recruited from amongst those who have trained in general hospitals. The estate surrounding this modern "hospital for the insane" consists in all of 343 acres, including all rights and interest in Bishop Loch, a fine sheet of water close to the new buildings.

### A USEFUL GIFT.

At the annual meeting of the Kettering Nursing Association the thanks of the committee were tendered to Mr. Taylor, who had been kind enough to place a bicycle at the disposal of the nurses. "It was felt that this would be the means of materially facilitating and lightening their work, especially in visiting patients in the outskirts of the town." We hope Mr. Taylor's example will be followed by other friends of district nurses about the country, to whom such a gift would be of untold value in scattered districts, where much of the nurse's strength is expended in long and weary tramps from case to case. The Kettering nurses are also to be congratulated upon their change to a new and better "home" during the past year, where every arrangement has been made for their comfort and well-being.

### A STEP FORWARD.

We note with much satisfaction that Mr. Chaplin, in his capacity as President of the Local Government Board, has consented to receive a deputation from the Workhouse Infirmary Nursing Association on Wednesday next, the 15th instant, at the House of Commons at half-past two o'clock. It will be devoutly hoped by all who are interested, not only in nursing matters generally, but in securing for the sick inmates of our workhouses all over the country the proper care and attention which is unattainable under the present system, that this step is, but the forerunner of sadly-needed revisions in the administration of infirmaries under the Poor Law. The Workhouse Nursing Association, by the energetic labours of its leaders, has done a great work in introducing by slow degrees trained nursing into institutions where "pauper help" was once considered all-sufficient, but matters have reached a standstill now, until the actual provisions of the Poor Law, framed before nursing, in any true sense of the word, existed, and now utterly out of date and impossible, can be intelligently altered to meet the requirements of the present day. What might be called the late outbreak of tragedies in workhouse infirmary wards has strongly arrested public attention and brought things to a climax, and it can only be trusted that Wednesday's deputation may be productive of reform in the very near future.

### MEDICAL WOMEN IN SCOTLAND.

Women students have been much to the fore in the ecent professional examinations for degrees in medicine and surgery in the University of Glasgow, and in the published lists of successful candidates the names of several ladies have figured prominently. At a recent meeting of the Govan Parish Council, a resolution making women eligible as medical officers under the Council was adopted, on the motion of Mrs. Greenlees. The chairman of the Medical Committee raised no difficulties in the way of the proposal, though one member of the Board doubted whether "general confidence would be felt in women medical officers" throughout the districts, and suggested that a better plan would be, in his view, for the Council to appoint instead a number of qualified midwives.

### WOLVERHAMPTON WORKHOUSE.

It is satisfactory to see that the Wolverhampton Board of Guardians have not allowed the grass to grow under their feet in commencing reforms in the nursing arrangements at the workhouse, the inadequacy in which was brought strongly into notice by the recent death of a patient from scalds received during the administration of a bath by a pauper wardsman. At the last meeting it was resolved, on the recommendation of the House Committee, to place the supply of water for the baths under the exclusive control of the nurse in charge of the wards; to draw up a new code of regulations for probationer nurses (a copy to be supplied to each nurse); and to place a thermometer in each bath-room. The adoption of the most important recommendation of all, *i.e.*, to increase the number of probationer nurses from eight to twelve, was postponed till the next meeting, when, it is to be hoped, this most needful addition to the staff will be assented to without hesitation. It is naturally of little use to make the charge nurse solely responsible for the bathing of the patients if she has, as stated, seventy-one sick people under her care. The only way to place matters on a proper footing is at once to increase the nursing staff, if necessary appointing another trained nurse instead of merely adding to the number of probationers.

### NURSING APPLIANCES.

Our readers will remember that the editors of *Nursing Notes* originated the idea of holding an exhibition of nursing appliances in London last autumn, when they showed a large number of articles of the greatest interest to nurses at the Trained Nurses' Club, 12, Buckingham Street, Strand. This exhibition proved so successful that its promoters have now decided to keep a selection of the most practical appliances, &c., permanently on view at the club. Nurses can thus examine them at their leisure, and will in this way be able to judge better of their respective merits than they could during the crowded time of the exhibition. The appliances are now on view daily (Saturdays excepted) from two till six. Members of the club may inspect them free of charge, while outsiders will be asked to pay the modest sum of threepence. Any contributions of practical nursing appliances will be gladly received and shown. All communications to be addressed Publisher, *Nursing Notes*, 12, Buckingham Street, Strand.

### TO HELP THE CHILDREN.

Mr. T. Glenton Kerr, the secretary of the North Eastern Hospital for Children, has sent us the first number of an interesting little magazine called "Children" which is to be produced quarterly in the interests of that most deserving institution. Hospitals for children always awaken a special interest, but when such institutions are buried away from the wealthy of the population their claims must be brought forward continuously, or their very existence might be forgotten save by those they so greatly benefit. The bright little paper before us strikes us as an attractive and novel method of appeal, and we wish the scheme all success. It is issued quarterly by the Children's Association, and does the editors great credit. The first number contains contributions from F. W. Robinson and John Strange Winter, and is illustrated by new and charming portraits of the children of the Duke and Duchess of Connaught and by drawings from the hand of Mr. A. Hartrick.

### RESPECTABILITY CONSTITUTING INELIGIBILITY.

To anyone conversant with the ways of the poor, it is strange to find the clothes in which they appear at a hospital are sometimes taken as a proof of their fitness or unfitness for free relief. Yet the possibility that out-patients might be disqualified because they looked too respectable was brought forward at a recent annual meeting. The injustice of such a test, unsupported by other evidence, is obvious to all who know the brave struggle for decent outward apparel, and the self-denial practised by the poor souls whose earnings are much less than those of the slatterns, whose claims to free advice and medicine are never questioned.

### SHORT ITEMS.

The second reading of the Midwives Bill is fixed for May 6th. It should be borne in mind by all who are interested in the progress of this measure, that they should use their personal influence by writing to, or seeing, their respective Members of Parliament, urging their attendance and support on that occasion.—The April number of "The Country House" contains an interesting article by Miss Gethen on the Deep Sea Mission to Sailors.—A memorial tablet has recently been unveiled in the Westminster Hospital chapel to the memory of one of the nurses who died in November last of scarlet fever, contracted while nursing in Luton during an epidemic of the fever. On the tablet is inscribed, "In loving memory of Margaret Anderson Allan, who died at Luton, Bedfordshire, in the faithful discharge of her duty, November 30th, 1895, 'He that loseth his life shall save it.'"—A successful concert has been given in Rochdale on behalf of the District Nursing Association in that town. A large audience gathered in the public hall on the occasion.—The Gainsborough Choral Union gave on Palm Sunday a performance of the cantata, "Daughter of Jairus," in aid of the local nursing association.—The next examination for the certificate of the Medico-Psychological Association of Great Britain and Ireland in nursing and attending on the insane will be held on Monday, May 4th. All particulars in reference to these examinations may be obtained from Dr. Spence, Burntwood Asylum, Lichfield.—At the spring meeting of the south-western division, to be held at Bath on April 14th, a discussion on "The Nursing Staff in Asylums" will be introduced by Dr. P. W. MacDonald.—The next lecture at the Midwives' Institute and Trained Nurses' Club will take place on April 17th at a quarter to eight o'clock p.m. Lecturer, Dr. Des Vœux; subject, "Collapse, Syncope, and Allied Conditions."—Dr. Adler, the Chief Rabbi, accompanied by Dr. A. Eichholz, visited the Chelsea Hospital for Women the other day, and inspected the provisions made for the treatment and dieting of Jewish patients, with which they were much pleased.

### Hygiene: For Nurses.

By John Glaister, M.D., F.F.P.S.G., D.P.H.Camb., Professor of Forensic Medicine and Public Health, St. Mungo's College, Glasgow, &c.

### I.—HYGIENE AND ITS BEARINGS—THE HOUSE WE LIVE IN, IN RELATION TO HEALTH, CONSIDERED GENERALLY.

Hygiene is that science which teaches of the preservation of the health of man and of the conservation and prolongation of life. The word is derived from "Hygeia," who was in the pagan mythology designated the goddess of health. She was the daughter of Æsculapius. Hygiene has a collective as well as an individual application; its precepts are alike valuable to man in the mass, as to man in the unit. In its collective application, since it deals with the lives of the inhabitants of a State—which are its most valuable assets—it is denominated State medicine. In respect also that it instructs in the principles of prevention of disease, it is often designated preventive medicine.

But, by whatever name it may be called, the object of hygiene is to enable us to live as healthily and as long as possible by forewarning us against those causes which make for disease, and thus to avoid them. It therefore deals with everything within, without, and around us which will prejudicially affect the natural tear and wear of the body. Thus, the house we live in, the food we eat, the coverings of our bodies, the air we breathe, and the water we drink, all come under our purview. Our relations to our neighbour, the schemes of drainage and water supply common to large communities, will also demand attention. But all of these points will be treated from the point of view of the nurse, in order not only that she should be a more intelligent nurse, but that also she may the better be able to observe the relationships which subsist between her patients and their surroundings.

In the succeeding articles of this series it is proposed to treat the subject both systematically and succinctly, and in this connection probably the most important subject to be first discussed is "The House we Live in, in its Relation to Health."

The importance of this is at once apparent when it is recollected that the average person spends at least half of his life-time indoors; consequently the home forms a very important factor in our lives. Its condition, from a sanitary point of view, exercises considerable influence on the health of the inmates. It may only be in the direction of impairing vitality, or it may be in the production of actual disease. Most people cannot command everything they would desire in their houses; economic considerations frequently forbid. The exigencies, too, of city life compel many persons to live near their work, amid surroundings alike uncongenial as they are unhealthy. Hence the site of the house, its surroundings, its relation to climatic conditions, to wind, and sunlight, are beyond their control. It will be, at least, certain that the more open the space is around a house, the more bountiful will be the supply of air and sunlight. A house in a hollow is likely to prove a more unhealthy house than one on a hill; here the subsoil conditions—the soil upon which the foundations are laid—become of importance. The former house is more likely to be damp than the more elevated one, for reasons which will be pointed out later. Again, the drainage of the latter house will be better than that of the former, since water runs from heights into hollows.

Again, the kind of house in relation to the neighbour is of some importance. The one-tenant, or self-contained, house is more independent of the careless neighbour than the house in a flat, or apartments in a tenement occupied by different holders. The careless neighbour in such circumstances as the latter would likely prove a grave menace to the others, for instance, in the spread of the infectious diseases of children. The housing of people has an influence *per se* on the health of a community. The denser the population in a given area the higher will be the death-rate, under ordinary conditions. This is abundantly proved from the experience of large cities. If this dense population be composed largely of the vicious class, the death-rate will still be higher than if a population of equal density were made up chiefly of the industrious working-classes. The average density of any given population is arrived at by dividing the total number of the inhabitants by the area covered by their habitations. Thus we speak of so many persons per acre, or square mile, or whatever the unit of space may be. The actual density is obtained by ascertaining the precise number of inhabitants living within a certain defined and measured area. The average density, therefore, will be no true guide, since the most densely and most sparsely populated districts are "lumped" together, as are also the most open with the most crowded spaces. So, too, has the number of rooms in a house an effect on the health of its inmates. Generally speaking, the smallest houses—that is, those of the smallest number of apartments—are occupied by those whose struggle for existence is the hardest. With such people it is all work and no play, and little time, and perhaps less care, is exercised in observing ordinary cleanliness. There is but the scantiest heed for infant life, and hence follows a high death-rate in children. The law of the survival of the fittest is in full operation here, for life at the extremes of age—childhood and old age—is held cheap. As the house increases in size these conditions gradually disappear until a point is reached when disease-producing factors have least chance to operate, because of the observance of the principles of personal hygiene, and of the attention given to the sanitation of the home. It is not to be wondered at, therefore, that from all classes of disease, and at all periods of life, the mortality rates increase as you descend the scale.

If we view houses from the point of view of nursing, we can also see why it is that such things should be. Nursing, worthy of the name, is absolutely impossible in houses of the poorer classes, and any kind of medical treatment is well-nigh valueless. Recoveries are to be attributed more to the native strength of the patient—otherwise called the *vis medicatrix naturæ*, *i.e.*, the healing power of nature—than to anything the physician or the nurse may do. In the larger houses, on the other hand, both medical and nursing treatment are afforded room to work, and the patients having been brought up in healthier conditions are better able to resist the operations of disease; hence more recoveries follow. From these causes, as well as from other physical and moral causes, there are higher death-rates in the poorer class than in the better class houses. Probably the most important factor in the health-conditions of any home, be it small or large, is the "elbow-room," that is, the superficial floor-space and cubic space at the command of the occupants. It has been assumed, hitherto, that the average-sized family occupied the classes of houses described, but it is obvious that in houses even of four or five apartments, the elbow-room may be not greater, indeed may be less, than in houses of smaller dimensions. This involves the question of the house-space in relation to the number of occupants. There is a minimum amount of space, both superficial and cubic, which each adult person must have in order that health may be fairly maintained. Economy prohibits the maximum. The Legislature has taken this subject into consideration in connection with the sanitary supervision of the smaller houses of certain populous places. The reasons for this largely depend on ventilation. The following tables illustrate the amount of space given to children in the schools of different countries, and to adults in the poorer classes of houses.

Table I.—Showing School Spaces in Different Countries.

| Country. | Superficial Floor Space in Square Feet. | Cubic Space in Feet. |
|---|---|---|
| Great Britain (Government Code) | 10 | 100 to 140 |
| London School Board ... ... | — | 130 |
| Dundee School Board ... ... | — | 152 |
| Canada ... ... ... ... | — | 240 |
| Belgium ... ... ... ... | 10½ | 160 |
| ,, Educational League ... | 17½ | 338 |
| Holland (average)... ... ... | — | 131 |
| ,, Haarlem (average) ... | — | 160 |
| Bavaria (children of eight years) | — | 137 |
| ,, (children of twelve years) | — | 197 |
| Dresden ... ... ... ... | 7 | 152 |
| Frankfort (Med. Society) ... | 15½ | 300 to 325 |
| Basle (Switzerland) ... ... | 15½ | 148 to 165 |
| Sweden (Primary schools) ... | 16 | 188 to 266 |
| ,, (Higher schools)... ... | 17 to 23 | 271 to 352 |
| America (New York City) ... | — | 88 to 105 |
| Columbia (Commission) ... ... | 15 | 210 |
| Massachusetts (State) ... .. | Demands that each pupil receive 1,800 cubic feet of warmed fresh air per hour. | |

Taken from a paper by the Author on "The Hygiene of Schools" in the *Sanitary Journal*, January, 1895.

Table II.—Showing Cubic Space Allowed by Law.

| Cubic space allowed by Law or Local Regulations. | Cubic space per head. | Authority. |
|---|---|---|
| Board schools under Education Act ... ... ... | 80 | Education Act. |
| Board schools (minimum under New Code)... ... | 100 | Education Depmt. |
| General school-rooms ... | 130 | London School Bd. |
| Graded schools ... ... | 117 | ,, ,, ,, |
| Dundee Board schools—newest ... ... | 152 | Dundee School Bd. |
| Canadian schools ... ... | 240 | Canadian Govmt. |
| Ordinary dwelling-houses in Glasgow and Edinburgh, in one and two-roomed houses—for adult... ... | 400 | Glasgow Police Authority. |
| Two children under 10 ... | 400 | Glasgow Police Authority. |
| Common lodging-houses ... | 240–300 | |
| Poor Law for healthy persons | 300 | |
| Poor Law for sick persons... | 850–1200 | |
| Barracks ... ... ... | 600 | Army Regulations. |
| Army hospital wards ... | 1200 | ,, ,, |
| ,, Huts (free ventilation) | 400 | ,, ,, |
| ,, Hospital ... ... | 600 | ,, ,, |

### The Difficulties of a Provincial Matron.

By a Correspondent.

The appointment has been obtained, and the late sister of a fine London ward, with all modern appliances and the perfection of picked nurses, finds herself exalted (?) to the matronship of from 120 to 140 beds in a provincial hospital.

She enters upon the strange land without knowledge of the thorns, vipers, evil winds that abound; but she has not been many days before she realises how different is the atmosphere.

Too often the charge nurses she finds either resent the new rule or are unworthy to be kept, so one of the first things is to make changes.

The advertisements bring numerous applications with glowing testimonials—from women who have just finished one year's probationership to women who have dodged about in the nursing world for ten, all seeking a "sister's" and (more important from their view) a comfortable post.

Perhaps matron thinks one of the old staff nurses from the hospital she has just left might be helpful, and she succeeds in getting one, but to be "sister" here does not satisfy the nurse's aspirations. She affirms that "staff nurse" in her big hospital is far preferable to sister in *this* place. She misses everything, grumbles at the cases, the operating theatre, the staff honorary and resident, so she returns, and matron takes the best (she hopes) from a training school which is not London or large North-country hospital.

One is pert. Cannot see why she may not be "Hail, fellow—well met" with her probationers. Another is ill-tempered, and draws comparisons between present and late hospital. In fact, things seem unworkable with the untrainable elements.

It won't do to change again just yet. The hospital will "get known" in an unpleasant manner, so a painful struggle commences. Insensibly, matron lowers her standard to the capacity of this new kind of charge nurse she calls, with a shudder, "sister."

It is appalling, after the order and quietness of her own large ward, to see and hear these nurses and probationers. They giggle, gossip, and work like third-rate shopgirls, with an occasional touch of the cheeky barmaid. All deeply resent being what they call "kept under."

The weekly committee, too, potter round and discuss the new matron with the nurses. They trot to her room to tell her "a patient has complained the potatoes were hard yesterday." Then the house surgeon or surgeons are very particular about their food, and say, "Oh, I say, Matron, don't you think you could change that cook? Our dinner really, was too bad for anything last night." And poor matron feels perhaps she is not quite up to the cook-housekeeper business yet.

Then it is so strange to feel *de trop* in the wards when the young house-surgeon stalks in. But her vulgar charges are "mistresses of the situation" here, so matron with an half-apologetic little smile goes out of the ward. She in her heart has a great contempt for doctors, nurses—often treatment.

One morning she makes a raid on the operating theatre, which she knows is as horrid as the rest of the arrangements, and hears, "What's the matron doing in here? She ought to be in the stores with her tea and sugar."

An angel might run away from the accumulation of duties for matron ought to be also housekeeper, storekeeper, linen-woman, and most of all probationers' sister, for she must look after and live with her 18 to 20 probationers. And oh, how different they are from those she knew so many years. These have opinions above everything, but mostly circling round matters which do not concern them.

The provisioning of so many doctors, patients, nurses, and servants is a thankless task. The head nurses openly despise the wholesome plain joint, and, sneering at the leg of mutton, grumble in a way as strange as it is new.

But a brave heart will carry a matron through the first year until the difficulties so real and constant are only a remembrance. The isolation, the lack of cultured, refined fellow-workers becoming to some extent less apparent, she may be so happy as to secure charge nurses so suitable that she no longer shudders at the absurdity of the name of "sister" as applied to them, and the hospital reaching a higher standard may receive a better, nobler class of women as probationers. But it will always be a strain without the *esprit de corps* and organisation of the large training schools to keep up a high tone and make the hospital worthy of its nursing certificates.

These outposts of the nursing world are so exposed that it needs more than average courage to hold them. The rigid economy, the lack of essentials, as once understood, is hard to live under.

### Trained Nurses' Clinic.

### I.—TRAINING—INTRODUCTORY.

The meaning of the word training is interpreted by the dictionary as teaching by practice or the art of education, and both these definitions may be aptly applied to the special form of training which is bestowed on the modern hospital and infirmary nurse. She undergoes a most elaborate preparation for the responsible calling which she has elected to follow, and besides the theoretical and practical knowledge which she in due course acquires, she needs natural fitness as well as taste for her chosen work.

Some years ago the title of trained nurse was indiscriminately bestowed on persons who, having undergone an infinitesimal amount of training in nursing, adopted a distinctive dress and the designation of Nurse. A woman who spent two or three years in any kind of hospital therefore ranked as a very superior person indeed, and she posed unquestioned as an experienced professional nurse, her competency in all branches of her calling seldom being doubted by any save the doctor. The latter himself carried out such details of treatment as could not be safely entrusted in the hands of women of this type, although belonging strictly to the nurse's department.

But even under such conditions many intelligent, industrious, conscientious women contrived to acquire considerable skill and eventual promotion to positions of responsibility. Their success was, however, due to individual perseverance, as they chiefly taught themselves, paying dearly for their hardly-won knowledge. The systematic teaching of the present day was altogether unknown at that date, being, in fact, the result of very gradual development.

It is almost impossible for those who have just completed a satisfactory course of three years' training to comprehend the contrast of their experience compared with their sometime predecessors. The wards offered the same facilities for the education of doctors and the training of nurses in those days, but the opportunities were utilised to but a limited extent by many who have lived to see these same wards become schools in which the art of nursing is systematically taught.

It has taken considerable time and even more tact and patience to evolve the present system out of that of the past, and the improvement is not even yet universal, but there is little doubt of its being eventually adopted in every civilised country.

Before passing on to the preliminary preparation of the candidates who desire to be received as probationers in hospitals, it may be as well to devote a little attention to the question whether such a course is really needful. By some it is considered that the three years' hospital training now inevitable for the would-be nurse ought to suffice, without the additional expenditure of time and money necessitated by either preliminary or post-graduate teaching. No doubt this would be the case save for two things—the first being the deficiency of most girls' training in the ordinary duties of domestic life; the second, the incompleteness of the present hospital course.

For example, when a candidate learns that she is as yet too young to be admitted as a probationer, she is seldom content to devote the intervening period to the acquisition of a thorough knowledge of sick cookery, which would include a study of the constituents of foods and kindred subjects. The general housework in an ordinary middle-class family is full of educational value to the future nurse, but she is seldom willing to take advantage of the chance of becoming a good manager of time, stores, and money, preferring rather to dream and talk of the more attractive splints and bandages with which the future will familiarise her. Needlework, too, is seldom tolerated by the candidate waiting to begin hospital life, for which it is nevertheless an essential qualification. "I always ask candidates if they can sew," remarked the matron of a training school, "they look so surprised, but I never take anyone who is not a good needlewoman. How can I expect the patients' clothes and the ward linen to be properly looked after by a young lady who could not even mend her own clothes? Besides that, how would the splints and ward appliances fare in such hands?"

Experience having thus shown that the majority of probationers are unhandy at common every-day duties which are essential to the comfort of the sick, it is desirable that they should devote a few weeks to acquiring knowledge of many details which might well have been previously learnt from a practical mother or from an intelligent upper servant.

Besides these homely duties some elementary acquaintance has to be made with the more scientific part of a nurse's work, and she is taught (what every woman of every class should know) the rudiments of hygiene and sanitation, which are matters which will probably be some day included in the education of all boys and girls.

One of the best features of the preliminary probationers' training consists in it being carried on before the introduction of the pupils to the bedsides of the sick, so that the latter are spared discomfort, whilst the new probationer is able to avoid exhibiting in the wards her initial awkwardness. Thus the patients are distinctly benefited by the latest addition to the education of the trained nurse.

The incompleteness of the present course of the hospital or infirmary training (which nevertheless has so many admirable characteristics) will probably disappear in the future. The need, or at any rate, the demand, for three or four years to be devoted to hospital training has often resulted in unwary probationers pledging themselves to remain the whole of their time in some small institution where the narrow limitations of their experience are unobserved by themselves, until too late—in some cases not until they have passed through part of their course. Yet the obvious injustice of describing three or four years of such work as complete training when neither fever, maternity, nor mental nursing is included in the course, must be brought painfully home to many who possess a certificate for medical and surgical nursing. The fact that another year or two must be devoted to special branches of nursing is an unwelcome revelation to the ambitious nurse.

There is no reason why, in process of time, a complete system should not be evolved by which two years in a good general hospital or infirmary, followed by two years divided up between mental, maternity, and fever nursing, should entitle a nurse to what might be justly called a certificate of complete training.

### Death in Our Ranks.

A sad gloom has been cast over St. Thomas's Hospital this Eastertide by the death of Helen Sutton Haig Brown, lately Sister of Alexander Ward, and daughter of the Rev. Dr. Haig Brown, head master of Charterhouse School. A memorial service was held in the hospital chapel on Easter eve at two p.m., the time at which the funeral took place at Godalming, when a large congregation assembled, amongst whom were many members of the hospital staff. The service was conducted by the Rev. J. Grant Mills (hospitaller), who gave a short address, in which he spoke of the esteem and affection Sister Alexandra had won for herself during her work at St. Thomas's, testified to by the unanimous request from one end of the hospital to the other that a memorial service should be held. She was beloved by her patients, and the secret of her success lay in her great unselfishness, the essence of Christianity. Her career was full of promise. She had just entered upon a fresh start in life—a well-deserved promotion—but her life, though short, would not have been in vain if her noble example produced in others greater devotion to duty and a more earnest resolve to "do all for Christ's sake." The service ended with the impressive playing of the Dead March in "Saul" by Mr. Moon, the hospital organist.

### On Certain Aspects of the Nursing Question as Seen in England and Germany.

By a Certificated Midwife.

### VI.—THE POLIKLINIK.

I did not enter myself as a pupil in the *Entbindungs-institut* at Dresden, as my object in going there was to gain some knowledge of women's diseases and their treatment. Attached to the hospital there was a large *Poliklinik*, or, as we should say, "out-patient department," though this does not really express the meaning of the German term. Many of the patients were taken in—all, I believe, if their cases required skilful treatment; and there were besides many beds specially reserved for paying patients who came to be operated on from all parts of Germany, Dr. Winckel (generally called the *Geheimrath*) and his assistant staff having a wide fame. This hospital was very much larger than that of Stuttgart, the number of pupils in the *Hebammen Schule* was about eighty. I was free to attend all lectures and witness any labours of special interest as presenting abnormal conditions, but I had no work to do in the wards. The gynæcological department was my study, and when the *Poliklinik* was held twice a week I was always present, and also at the different operations which took place during my three months' stay. The *Geheimrath* resided with his family and staff of assistants in one wing of the institution, which stood in a lovely garden. Here also were rooms for young medical men, who came from all parts of Germany and various foreign countries to gain experience under the celebrated Professor. From six to twelve generally accompanied him when he made his rounds of an afternoon; and, owing to the introduction I brought with me from Dr. Fehling, I was allowed to be one of his followers, and even to take my turn in examining, and receiving his instructions in respect of diagnosis. I believe there was some special "foundation" for meeting the expense of boarding and lodging these foreign students, but it was limited to such as had taken out their qualifications as medical men (or women), and therefore I had no claim on it. But by favour I was granted a room and a share in their privileges, and only had to pay a small sum for my food, which I was allowed to draw from the kitchen that served the private patients. And I may here note that it was excellent, and that I have often wished for the plate of the *Bier-kalte-schale* or *Kirschen suppe* which used to be put on my table there.

The wing in which the *Poliklinik* was held was on the opposite side to that in which the staff resided; and while the free patients had the ground floor, the paying ones occupied the two upper storeys, and the centre of the building was given over to the school and the lying-in wards. In the same beautiful garden stood the general hospital, and also a lunatic asylum. The former had been a palace, and in it was shown the rooms inhabited by Napoleon during the siege of Leipzig. This accounted for the splendid garden, with its fountains and statues, which was thrown open to the public on Sundays.

The instruction given to the school was virtually the same as that at Stuttgart, and therefore calls for no comment. I attended most of the lectures out of pure interest, but I did not go in for any certificate. What I really gained at Dresden was some insight into the nature and causes of women's and children's diseases, and the antiseptic methods of treatment. The personal care of the staff of doctors with regard to the details of the latter, and the washing and disinfecting which they practised in the wards filled me with astonishment and respect. And under Fraülein Weber's rule in the labour ward, every scholar had to carry out like precautions, and even to present her hands for minute inspection each morning, as well-cut nails and perfect soundness of skin were insisted on before they were allowed to assist at a confinement. I often thought of what I used to see in English hospitals—the careless dipping of tips of fingers into a tiny basin of water faintly tinged with Condy, and the sheets of cotton-wool presently wanted for an operation case left airing before the fire in a ward where there were patients suffering from all sorts of suppurating wounds. This was many years ago, and I know things are different now. But I was fresh from such scenes then, and when I saw the minute supervision which was given to the preparation of a room and the necessary appliances when an operation was to take place under Dr. Winckel's auspices, I learned a lesson which I never forgot, and did not wonder at the results he obtained.

But my reflections were sometimes of another nature. I noticed in Germany, and especially in Dresden, an unusual number of ricketty children, and as I walked in the streets I have sometimes counted the working-men as I passed them, and noted that one out of every three would have crooked legs. It used to make me very angry when I was told, "Yes, the *Englische-krankeit* is very common here." "Why the English disease?" I would ask. "You don't see our men like that; we take better care of them when they are babies." I got no satisfactory answer to my query, but my thoughts ran on the superiority of my own country. A paternal Government can do much in the way of training and teaching, but it cannot follow the mothers into their homes and insist on regulations being carried out. The truth is, I think, that Germany is too poor to bring up the number of children which it produces, and the heart of the woman waxes faint under the heavy load of life. Few can suckle their own babes, because of their field and factory labour, and fewer still can afford to buy milk for them. So they are fed on meal compounds, against which the medical men inveighed with but little success when I was there. I quote from one of their own books the following: "The mortality amongst our infants is a common scandal, in which each part of our Fatherland bears its share of blame. With the arm of the law nothing can be effected. The mother who does not suckle her child cannot be punished, nor can the police stop the bottles of pap, or the use of *Schlotzer* (rags with bread and sugar tied up and thrust into crying mouths), so commonly seen all through the land." In England we have our Factory Act, which does more good than is generally realised in keeping the mothers from work which necessitates the neglect of helpless babes. And, moreover, we are free from the awful burden of *Militärdienst*, which takes every fit man from the cultivation of the soil during some years of his life, and leaves it to the women, who, swaddling their infants in pillows and thrusting a *Schlotzer* into their mouths, go to plough and reap and drag carts, and grow prematurely old under the excessive toil. And the taxation is also enormous to keep up the army and the executive of the empire, so that their earnings do not suffice to procure nourishing food, and disease of the bone is frequent.

I went to Germany to learn, as I set forth at the beginning of these articles, and among the various knowledge I acquired (and which I shall ever prize) stands clear in my mind the conviction of the supremacy of English children over their cousins across the Channel. And I congratulate their mothers from the bottom of my heart, for never could I hear from their lips what I once heard from a German parent, with whom I condoled on an accident which crippled her child's hand for life. "It is nothing to grieve over, my Fräulein; it will save my boy from having to serve in the army." A paternal Government in England! Heaven forefend, if that is what it means.

### Dress and Uniforms.

By a Matron and Superintendent of Nurses.

### POPLAR HOSPITAL FOR ACCIDENTS.

Few who visit the Poplar Hospital for Accidents can fail to be impressed with the neat and appropriate uniform worn by the nursing staff. The exceptionally pleasing group we have the privilege of reproducing owes much to the extreme kindness and courtesy that placed every facility in the way of our artist, and for which we must take this opportunity of expressing our gratitude. The illustration depicts a ing to the throat, which is elongated at the shoulders into straps which cross behind, and fasten on to buttons at the waist. The chief point to be attended to in aprons of this shape is the cut, as they relieve by contrast the dark and sombre hue of the dress, and when well-fitting are very becoming to the wearer. Turn-down linen cuffs and collars relieve the dress at the back and wrists, and the costume completed by a pretty cap of the Sister Dora shape in

·Sister· ·Probationer· ·Nurse·

sister, staff nurse, and probationer, whose attitudes indicate that smartness and alertness which we are wont to associate with only the best type of nurse. The "sister" who occupies a seat at an escretoire is engaged apparently in receiving reports from her assistants. Her dress is of navy blue drill, plainly made, and full in the skirt. The bodice buttons in front, and is fastened to the skirt by a band which encircles the waist. We have before expressed our approval of the principle of washing dresses for ward sisters, the hygienic advantages of which are enormous over the more popular woollen material, which is only discarded when worn out.

The apron is of fine white linen, and has a high bib, reach- white cambric, which ties in a neat little bow under the chin. A narrow-striped blue and white galatea is worn by the staff nurse, which is made in the conventional style, and looks clean and workmanlike. The apron resembles in shape that worn by the sister, and presents a very dainty appearance over the fresh-looking material beneath. The cap is similar also, with the exception of being without strings.

The probationers' dress is blue and white of a much wider stripe, to distinguish her from the staff nurse. Over this is worn a white linen apron, with a short bib, and straps that cross behind and fasten at the waist. The cap is the Sister Dora, without strings. White linen cuffs and collars complete the appropriate and attractive costumes in both instances.

### A Book and its Story.

### "A MONK OF FIFE."*

It is impossible to compress within the limits of a single page an adequate criticism, or, indeed, anything more than a hasty and imperfect account of this singularly attractive volume, which has achieved the rare distinction of running through four editions within the space of two months. And the reason of its popularity must be apparent to everyone who has sufficient historical knowledge to appreciate the amount of conscientious labour which Mr. Lang has bestowed upon his work. The story is a romance of the fifteenth century. Norman Leslie, son of Archibald Leslie, of Pitcullo, a cadet of the great house of Rothes, is a student at the University of St. Andrews in the autumn of 1428. His father had wished to make a Churchman of him; but, as he says, he took more pleasure in sports and pastimes than in divine learning. Like other students, he was fond of golf, "that wicked game of the Golf, now justly cried down by our laws, as the mother of cursing, idleness, and mischief, of which game, as I verily believe, the devil himself is the father"; and one day, while playing "at this accursed sport," he quarrels with his opponent, knocks him down with "the iron club," and, supposing him to be dead, leaps into a boat, and pulls out into the Eden, the tidal river which runs into St. Andrews Bay. He sails to Berwick, and from thence makes his way to Bordeaux.

We have somewhere read that in those days the favourite toast of the Scottish soldiers of fortune was "Peace at home and plenty wars abroad"; and though Norman Leslie will probably never become so famous as Quentin Durward or Dugald Dalgetty, yet he is worthy to be ranked as a younger brother of those illustrious heroes. At a period subsequent to that of Mr. Lang's story, Europe was, as is well known, overrun by those hardy adventurers, and in many countries the desperate valour of the Scots won battle after battle; but it was to France that a Scottish gentleman naturally found his way, to France, where the Garde Ecossaise were held in such high honour that their captain stood beside the King at his coronation, and received the Royal robe as his due; where, when a fortress was captured, the keys were handed to them; where, at all times and seasons, in the privacy of the palace or during public ceremonies of State, six gentlemen of their number, clad in white coats overlaid with silver lace, token of their fidelity, stood always next the person of the king. At no time were the Scots more popular in France than when Mr. Lang's hero landed at Bordeaux. It is among episodes such as this that Mr. Lang takes us in the "Monk of Fife"; and perhaps it may appear to some that the details of the fighting, the assaults, the sorties, the ambuscades, are too fully described. But the book would have been altogether wanting in historical verisimilitude if, purporting to come from the pen of one who fought by the side of Joan of Arc, it had not contained minute particulars of even insignificant skirmishes. At the period where this story begins it seemed as if France was soon to become a mere province of England. As Norman Leslie, now on his way to Orleans, hurries through a country all desolate from the ravages of war—hoping that he will reach his destination before the city falls—he comes upon Noirouple, a Cordelier Friar, who is standing in the water of a stream which he cannot cross because the bridge has broken down. "His Cordelier's frock was tucked up unto his belt, his long brown legs were naked. He was a huge, dark man, and when he turned and stared at me, I thought that, among all men of the Church and in religion whom I had ever seen before, he was the foulest and most fierce to look upon. He had an ugly, murderous visage, bold eyes and keen, and a right long nose, hooked like a falcon's." And thenceforth throughout the rest of the story this friar is Norman's evil genius. "This encounter was the beginning of many evils."

They join a party of wanderers, in company with whom they journey until near Chinon, where the Court was at that time. About five miles from the town they come to a wood. "There was not a white coin among us; night was falling and it seemed as if we must lie out under the stars and be fed like the wolves we heard howling in the wind." While the starving band are talking, they suddenly hear the sound of horsemen approaching, and their leader orders his party to lie in ambush.

Young Leslie, however, refuses to act as a common "cut-purse, and is about to cry '*A secours*,'" when, quick as lightning, the Cordelier seized him, crammed a napkin into his mouth, so that he could not cry, trussed him with a rope, and kicked him into the bottom of the ditch. The ambush was prepared; but the riders had taken the precaution of sending one of their number in front, to see if the way was safe. So the strangers passed in safety, talking in the darkness to each other. "On the night there rang a voice, a woman's voice, soft but wondrous clear, such as never I knew from any lips but hers who then spoke; that voice I heard in its last word, 'Jhesus!' That voice said, 'Nous sommes presqu' arrivés, grâce à mes Frères de Paradis.' . . . On the forest boughs above me, my face being turned from the road, somewhat passed, or seemed to pass, like a soft golden light, such as in the Scot's tongue we call a 'boyn,' that oftentimes, as men say, travels with the blessed saints."

The Cordelier departs with his band of ruffians, leaving Norman gagged and bound in the wood, where he is till morning. Then he hears voices. "These, like the others of the night before, came nearer, and a woman's voice gaily singing. And there awoke such joy in my heart as never was there before, and this was far the gladdest voice that ever yet I heard, for, behold, it was the speech of my own country."

The travellers prove to be Elliot Hume, a Scottish maiden, and her father, who are on their way to Chinon. They rescue Norman, and, as they are going along, he hears from them of the "famed Purella, the Laravian peasant lass, who is to drive the English into the sea." This holy thing can have been no other but that blessed maiden, guarded by the dear saints in form visible, whom this gentleman, for the sin of keeping evil company, was not given grace to see.

Thereafter, with occasional separations, the fortunes of Norman Leslie are united with those of his new-found friends, while the central figure of the romantic Jean d'Arc, on whom Elliot Hume attends, and for whom Norman Leslie fights, till the last scene of all. The hero, after attempting to rescue her from prison, is in Rouen. "I stood afar off at the end, seeing nothing of what befell; yet I clearly heard, as did all men then, the last word of her sweet voice, and the cry of 'Jhesus.'

"Then I passed through the streets, where men and women, and the very English, were weeping, and saddling my surfleet horse, I rode to the east port. When the gate had closed behind me I turned, and, lifting my hand I tore the cross of St. George from my doublet. 'Dogs!' I said, 'ye have burned a saint. A curse on cruel English and coward French. St. Andrew for Scotland!' The shafts and bolts hailed past me as I wheeled about; then there was mounting of steeds and a clatter of hoofs behind me, but the sound died away ere I rode into Louviers. Then I told them the tale which was their shame, and so betook me to Tours, and to my lady." How Leslie marries Elliot Hume, and how, after a short wedded life she dies, and he, "leaving France, retires to the Abbey of Dumfermline," must be read in Mr. Lang's own words.

* "A Monk of Fife." Andrew Lang. (London: Longmans. 1896.)

### Novelties for Nurses.

SPRING NOVELTIES.

Signs are not lacking of the immediate approach of Spring. Around us the trees are beginning to bud, and the song of the blackbird and thrush announce the awakening of Nature from her winter's sleep. The recent spell of balmy weather tempted many of us, no doubt, to discard our heavy clothing, regardless of the time-honoured proverb,

"Until May is out,
Cast not cloth nor clout."

The cold winds and disagreeable showers that followed proved the wisdom of this advice; but April warns us that the change can be but temporary, and bids us prepare for brighter days. Mr. Egerton Burnet, of Wellington, Somerset, is as usual in the van of fashion with a charming assortment of materials adapted for the season. We notice among a singularly choice collection some lovely soft shades in grey serge and alpaca. The latter has a silk finish that is very attractive, and would make up into an extremely pretty, as well as a useful, costume. A shade in iron grey would also look very stylish. "The Loire," a new silk and wool mixture, calls forth special admiration. It is a combination of black and white tastefully blended together, an ideal fabric for those in half-mourning. The tweeds and serges, of which there is an endless variety, leave nothing to be desired, and sustain the deservedly high reputation maintained by this firm for the manufacture of these special goods. A fine cloaking in navy blue or black will be found useful, and has a durable appearance. This is especially worthy the notice of any nurse who may be wanting a light summer cloak. It would also make up into a desirable uniform dress, and look very nice. The ginghams and other washing materials are prettier than ever this season; the former are always cool and light looking, and the many delicate and beautiful shades in which they can be obtained make them always popular. They are, moreover, marked by a simplicity which eminently adapts them for use in the sick room, while the extremely low price at which they are sold places them within the reach of all. The tailoring department recently added to the establishment is a great convenience to customers selecting a pattern and wishing it made up on reasonable terms, and without the trouble entailed by going elsewhere. Self-measurement forms are supplied to facilitate matters and save unnecessary delay. Our readers will be well advised if they send for patterns before the rush comes, as it is sure to do a little later on. They will not be disappointed with the excellent quality and variety of the patterns submitted. Any length is sold, and carriage is paid on all parcels exceeding 20s. in value.

EAU DE COLOGNE.

The very best eau de Cologne we have yet come across is that called "4711." Once used in the sick-room or otherwise it will distance all rivals, we feel sure, as it certainly deserves to do. It can be obtained of most vendors of perfume, and the central depôt is at 62, New Bond Street.

### Appointments.

Royal Bath Hospital and Rawson Convalescent Home, Harrogate.—Miss Eleanor A. Waters has been appointed to the post of Matron at this institution. Miss Waters was trained at St. Mary's Hospital, Paddington. She has since worked as sister at the Royal Naval Hospital, Chatham, as sister of the boys' ward at the Margate Infirmary, and held the appointment of matron at the Ramsgate Convalescent Home of St. Luke's Hospital for Mental Cases, London. Her testimonials are excellent. Miss Waters has our best wishes for all success in her new sphere of work.

### Everybody's Opinion.

FEVER NURSING.

"Ariadne" writes: I have been reading your article in The Hospital "Nursing Mirror" for March 28th, on "Wanted, Good Fever Nurses," and the only wonder to me is that it did not appear several years ago. I have had a long experience of fever nursing as probationer, nurse, and matron, and I think the reason why the Metropolitan Asylums Board hospitals cannot get good nurses is easily seen by one who has nursed in one of their hospitals. It is true the pay and the food will bear comparison with other hospitals, both being good. But when a probationer has to work thirteen hours a day six days in the week, and is only off duty one half day in the week and once or twice in the week after eight o'clock supper, you will imagine that few girls of the better classes would care to enter for training. But the good salary allures many girls of the servant class who think it the correct thing to promenade the streets between eight and ten p.m. These girls form by far the larger portion of the fever hospital probationers, and their general conduct and their conversation at meals is so, let us say, unrefined, that if a well-brought-up girl goes to one of these hospitals as first or second assistant nurse she usually only stays quite a short time. It is possible one might stand the very hard work having pleasant co-workers; or the unpleasant company, if the work were not quite so hard and the time off duty at more suitable hours; but to have to endure both is more than most refined women can put up with.

PRIVATE NURSING BY THE DAY OR HOUR.

"Nurse J." writes: I have read with much interest the letters under this heading. I have been doing daily work for the last three years, and it has been well received by the doctors. Financially it is not so good as private nursing, but if one has private means, or can live at home, a fair income may be made.

Miss J. C. Wood writes: In reply to "Trained Nurse," I would say that the "Daily Nurse" has been worked from this hostel for the last five years, and on the whole with success. It has certainly met a great want. As to the financial side of the question, I doubt if a nurse could make it sufficiently remunerative if living by herself; as one among a number, it can be worked at a small profit. I know of two nurses who severally tried it and did not find they could secure enough regular work to pay their expenses in London. A nurse in Hampstead is working on these lines, and I believe quite successfully.

### Where to Go.

Royal British Nurses' Association.—A meeting of the General Council will be held at 17, Old Cavendish Street, W., on Friday, April 10th, at 5 p.m.

Queen's Hall, Langham Place, W.—An afternoon concert will be given at Queen's Hall on Saturday, May 2nd, at three o'clock, in aid of the funds of St. Thomas's Hospital. The Prince of Wales and the Duke of York are amongst the patrons, and the Duke and Duchess of Connaught intend being present. The vocalists will include Miss Esther Palliser and Mr. Andrew Black. Tickets, 10s. 6d., 7s. 6d., 5s., and 2s. 6d., may be obtained of the Treasurer, St. Thomas's Hospital, S.W.

### Presentation.

On Easter Day, Dr. Wilson, the medical superintendent of the Croydon Infirmary, Thornton Heath, was presented with a handsome testimonial in the shape of a gold watch, with suitable inscription, from the united staff of past and present officers at the infirmary, as a small token of their respect and esteem. The presentation was made by Nurse Bell, the oldest resident nurse, and accompanied by a kindly letter of greeting from the contributors.

### Minor Appointments.

Buckinghamshire General Infirmary, Aylesbury.—Miss Annie Bullock has been appointed Charge Nurse at this institution. She received her training at the Salop Infirmary, and has since worked at the Durham County Infirmary.

Bath Statutory Hospital for Infectious Diseases.—Miss D. Robinson has been appointed Head Nurse at this hospital. She received her training at the North-West London Hospital, and has since worked as charge nurse at the Chelsea Infirmary, and of the Medical Pavilion, Sheffield Infirmary, her last appointment having been as superintendent nurse at the Bath Workhouse Infirmary.

Isolation Hospital, Nottingham.—Miss Alice Turney has been appointed to the post of Sister at this hospital. Miss Turney was trained at the Bradford and Nottingham General Infirmary. We wish her all success in her new work.

### Notes and Queries.

### Queries.

(5) *District Nursing.*—Can you tell me of any District Nursing Association which trains nurses without a premium?—*Nurse Harriette.*

(6) *Uniform.*—Will you tell me if I can claim my second year's uniform when leaving an institution at the end of a year and eight months? I have been told that after six months it can be claimed as part of the salary.—*O. W.*

(7) *Monthly Nursing.*—Can you tell me if there is any institution in London or country where a monthly nurse would find employment at a small salary?—*Nurse Alice.*

(8) *Male Nurses.*—Will you tell me how I can become a fully-trained male nurse? I should not object to pay if needful, and have a great desire to be trained before going out to Africa, as I hope to in a few years' time.—*Chemicus.*

(9) *L. O. S. Diploma.*—(1) Would it be possible to take the L. O. S. diploma during six or eight weeks' holiday in June, and can you tell me the fees? (2) Will you also kindly recommend a good book on midwifery?—*Nurse.*

(10) *Nursing in the U.S.A.*—Can you advise me how I can obtain admission as probationer into one of the large hospitals in the United States? My people live in Montana, and I should like to be nearer home than England during my training.—*M. W.*

(11) *Flat-foot.*—Can you tell me where I can obtain Holland's instep arch? I am a great sufferer from flat-foot.—*Nurse M. E.*

### Answers.

(5) *District Nursing* (*Nurse Harriette*).—Get "How to Become a Nurse" (Scientific Press, 428, Strand, W.C.), and read the chapter on district nursing. Apply to the associations there mentioned.

(6) *Uniform* (*O. W.*)—It will depend upon the terms of your agreement with the institution. In some cases a certain amount of uniform is supplied "when required," in others a definite number of dresses, aprons, &c., are given "each year." In the latter circumstance you would probably be entitled to at all events some portion of the second year's uniform. Have you spoken to the matron on the subject? She will be the proper person to apply to in the first instance.

(7) *Monthly Nursing* (*Nurse Alice*).—The Secretary of the Midwives' Institute, 12, Buckingham Street, Strand, might help you with advice, if you write enclosing stamped envelope, and stating qualifications.

(8) *Male Nurses* (*Chemicus*).—Unfortunately, there is no hospital in England where arrangements have been made to train men as nurses. You might write to the Hamilton Association for Providing Trained Male Nurses, but we do not know any particulars as to how the training is obtained.

(9) *L. O. S. Diploma* (*Nurse*).—(1) Write to the Secretary of the L. O. S., 54. Berners Street, London, W. (2) "Practical Handbook of Midwifery," by Haultain, 6s. (Scientific Press, 428, Strand). Your appreciation of The Hospital is very pleasant. We are glad you find it so helpful.

(10) *Nursing in the U.S.A.* (*M. W.*)—"Burdett's Hospitals and Charities" (Scientific Press, 428, Strand, W.C., will give you the names and addresses of all the chief nurse training schools in America. Write direct to the matrons whose names are given in each case.

(11) *Flat-foot* (*Nurse M. E.*).—We do not know of Holland's Instep Arch, but we can recommend Pond's "Arch-support Socks," to be had from the maker, J. Pond, 23, Castle Meadow, Norwich, or at the London agents', the London Shoe Company, 45A, Cheapside, E.C.

### Wants and Workers.

[The attention of correspondents is directed to the fact that "Helps in Sickness and to Health" (Scientific Press, 428, Strand) will enable them promptly to find the most suitable accommodation for difficult or special cases.]

*A Doll's House.*—The matron of the Sisters' Home, Charleville Circus, Sydenham, S.E., writes: Would anyone like a doll's house? I know of a lady who would be glad to give one to any home for sick children or hospital where they would like it. It is not new, but is in good preservation, and she would be glad to send it by Carter Paterson to any place in London if she knows where it would be appreciated.

### For Reading to the Sick.

### EASTER.

**Motto.**

"Lo, the winter is past."—*Song of Solomon* ii. 11.

**Verses.**

One Easter morn, one spring eterne,
Where lilies never fall,
Where mystic stars in order burn
Round Thee—the Lord of all!
—*Frederick George Lee.*

Rise, heart! Thy Lord is risen! Sing His praise
Without delays,
Who takes thee by the hand, that thou likewise
With Him may'st rise;
That, as His Death calcinèd thee to dust,
His life may make thee gold, and much more just!
—*Herbert.*

'Tis the spring of souls to-day,
Christ has burst His prison;
And from three days' sleep in death
As a sun hath risen! —*Neale.*

Arise, sad heart, if thou dost not withstand
Christ's Resurrection thine may be;
Do not by hanging down break from the Hand
Which, as it riseth, raiseth thee:
Arise! Arise! —*Herbert.*

Sometimes a light surprises
The Christian while he sings!
It is the Lord who rises
With healing in His wings.
When comforts are declining
He grants the soul again
A season of clear shining,
To cheer it after rain.

In holy contemplation
We sweetly then pursue
The theme of God's salvation,
And find it ever new;
Set free from present sorrow,
We cheerfully can say,
E'en let the unknown to-morrow
Bring with it what it may!
—*William Cowper.*

**Reading.**

"I am come that they might have Life, and that they might have it more abundantly."

"Christ being raised from the dead dieth no more."—*Rom.* vi. 9.

Even this present life is full of the rhythm of the Resurrection; it is ever ready to remind us of the news of Easter. Time after time, if we will have it so, as we look at the visible world, as we gain or recall the lessons of experience, we see some rendering, as it were, of the glory of this Queen of Feasts, some parable of the empty tomb and the stone rolled back and the triumphs over death. When the day breaks and the shadows flee away, and all life stirs and wakes again; when the long tyranny of winter yields, and the flowers appear upon the earth, and the time of the singing of birds is come; when some great sorrow, or anxiety, or mood of sadness passes from our hearts, and we re-discover the reality of joy . . . As Nature would prophesy of the Resurrection, and show faith in outward signs what Easter means, her voice, her power, falters; she can but prophesy in part, for she has no form or type in all her wealth that will serve to tell of Him who "being raised from the dead dieth no more." . . . Here is the unique, distinctive splendour of our Saviour's triumph; here He leaves behind Him every earthly semblance of His Resurrection, "He dieth no more."—*Dean Francis Paget.*

## Figures and Tables

**Figure f1:**